# T_REG_king From Gut to Brain: The Control of Regulatory T Cells Along the Gut-Brain Axis

**DOI:** 10.3389/fimmu.2022.916066

**Published:** 2022-06-30

**Authors:** Juli Choi, Bo-Ram Kim, Begum Akuzum, Leechung Chang, June-Yong Lee, Ho-Keun Kwon

**Affiliations:** ^1^ Department of Microbiology and Immunology, Yonsei University College of Medicine, Seoul, South Korea; ^2^ Institute for Immunology and Immunological Diseases, Yonsei University College of Medicine, Seoul, South Korea; ^3^ Brain Korea 21 PLUS Project for Medical Sciences, Yonsei University College of Medicine, Seoul, South Korea

**Keywords:** regulatory T cell, microbiota, gastrointestinal tract, central nervous system, gut–brain axis, neuroimmune

## Abstract

The human gastrointestinal tract has an enormous and diverse microbial community, termed microbiota, that is necessary for the development of the immune system and tissue homeostasis. In contrast, microbial dysbiosis is associated with various inflammatory and autoimmune diseases as well as neurological disorders in humans by affecting not only the immune system in the gastrointestinal tract but also other distal organs. FOXP3^+^ regulatory T cells (Tregs) are a subset of CD4^+^ helper T cell lineages that function as a gatekeeper for immune activation and are essential for peripheral autoimmunity prevention. Tregs are crucial to the maintenance of immunological homeostasis and tolerance at barrier regions. Tregs reside in both lymphoid and non-lymphoid tissues, and tissue-resident Tregs have unique tissue-specific phenotype and distinct function. The gut microbiota has an impact on Tregs development, accumulation, and function in periphery. Tregs, in turn, modulate antigen-specific responses aimed towards gut microbes, which supports the host–microbiota symbiotic interaction in the gut. Recent studies have indicated that Tregs interact with a variety of resident cells in central nervous system (CNS) to limit the progression of neurological illnesses such as ischemic stroke, Alzheimer’s disease, and Parkinson’s disease. The gastrointestinal tract and CNS are functionally connected, and current findings provide insights that Tregs function along the gut-brain axis by interacting with immune, epithelial, and neuronal cells. The purpose of this study is to explain our current knowledge of the biological role of tissue-resident Tregs, as well as the interaction along the gut-brain axis.

## Introduction

**Figure 1:**
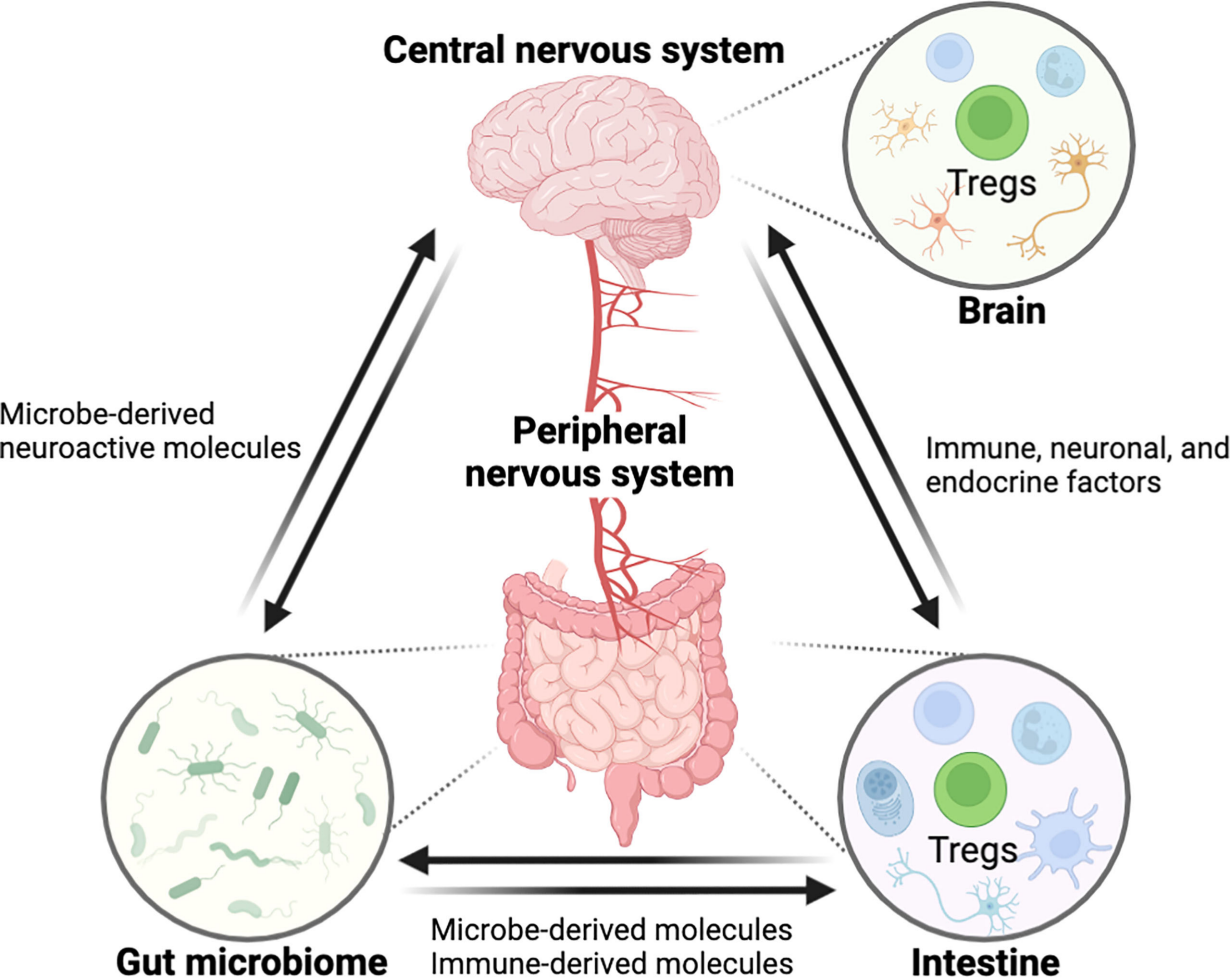
Schematic overview showing the interaction between the GI tract and the CNS through the ‘gut-brain axis’. The central nervous system is connected with the intestine by the peripheral nerves for reciprocal interaction and tissue homeostasis in which immune cells including Tregs are involved in this entangled inter-organ communication. Immunological factors, produced by gut immune cells, can regulate the nerves innervating to the intestine, eventually affecting the CNS function. In the opposite direction, neurological factors such as neurotransmitters can act on the gut immune system. Furthermore, the gut microbiome can modulate the gut-brain axis by microbe-derived molecules. In these processes, Tregs may act as a critical regulator of pathophysiology along the gut-brain axis. (All figures in the review were created with BioRender.com).

The gastrointestinal (GI) tract and central nervous system (CNS) are constantly in communication with one another through a bidirectional link termed as the ‘gut-brain axis’. The gut-brain axis is a complex inter-organ communication network, comprised of CNS, the peripheral nervous system (PNS), the intestinal immune system, and commensal microbiota that contributes to the regulation of CNS function, development, and host behavior (**Figure 1**) (1). The human intestine contains a wide and diversified microbial ecology, in which the microbiota has enormous effects on host’s physiology and pathology, implicating its roles in health and disease (2). The gut microbiota is crucial for the physiological function of the host including food digestion, development, protection against pathogens, and immune system education (3–5). In contrast, microbial imbalance (dysbiosis) in the gut has been related to several inflammatory disorders such as inflammatory bowel disease (IBD) and various peripheral autoimmune diseases (6–9). Importantly, numerous studies have also shown a link between dysbiosis in gut microbiome and a variety of neurological disorders, suggesting potential of intestinal inflammation by dysbiosis for neuropathology through modulation of the gut-brain axis (6, 10). However, despite recent advances in the field (11–16), little is known about how signaling from the gut microbiota to the brain governs CNS pathophysiology, and vice versa and the mechanisms underlying these complex interactions require elucidation.

Forkhead box P3 (FOXP3) ^+^ regulatory T cells (Tregs) are a subset of CD4^+^ T cells that operate as a checkpoint for immunological activation and are required to prevent systemic autoimmunity. The major function of Tregs in the intestine is to regulate inflammation. While Tregs can be recruited from the thymus to the intestine, the majority of Tregs in the gut are peripherally differentiated from FOXP3-negative conventional CD4^+^ T cells in order to induce tolerogenic responses to microbiota and dietary antigens (17, 18). Notably, the dysregulation of Tregs has a role in the development of chronic inflammatory disorders such as IBD (18, 19). Along with regulating immunological tolerance in barrier tissues, Tregs are critical for tissue homeostasis and remodeling in other organs, including the CNS, which has long been considered an immune-privileged site (**Figure 1**) (20, 21).

Recent research demonstrated that Tregs interact with a diverse range of resident cells in the CNS, resulting in a powerful neuroprotective effect in neuronal diseases (22, 23). The CNS-resident Tregs participate in controlling the neuroinflammatory response and neuroplasticity, associated with ischemic stroke, Alzheimer’s disease (AD), and Parkinson’s disease (PD). However, the characteristics of CNS-resident Tregs are poorly understood since the limited numbers of Tregs in CNS under homeostatic conditions (20, 24). Despite the established function of Tregs in each specific organ, the numerous mechanisms along the gut-brain axis remain poorly understood in health and disease. Thus, increasing our understanding of tissue-resident Tregs activity in the settings of inflammation and homeostasis may help improve therapy options for persons suffering not only from inflammatory disorders in barrier tissues, but also from neuroinflammatory illnesses.

This article discusses the induction, maintenance, trafficking, and activity of Tregs to maintain homeostasis in non-lymphoid tissues (intestine and brain) and suggest Tregs as the critical regulator of immune homeostasis along the gut-brain axis.

## Tregs in the Intestine

The intestine, including the small intestine and the colon, is the largest immune organ which is responsible for food digestion, nutrient absorption, and protecting the host against harmful pathogens while maintaining immune tolerance to innocuous microbial or dietary stimuli (25). Tregs found in the intestine differ from those residing in other tissues with tissue-specific characteristics and activities. While Tregs in lymphoid organs mainly express self-antigen specific T cell receptors (TCRs), substantial number of intestinal Tregs have a set of TCRs specific for intestinal antigens which is essential to suppress immune responses against harmless dietary antigens and commensal microorganisms (26, 27). Intestinal specific cues have potential influence for the development, migration, and maintenance of Tregs in GI tract (28). Certain microbiota members, in particular, supply antigens and immunoregulatory small molecules that affect intestinal Tregs on a constant basis (28). Thus, understanding the development and maintenance of intestinal Tregs reveals critical information regarding host-microbiota interactions in health and disease context (28).

**Figure 2:**
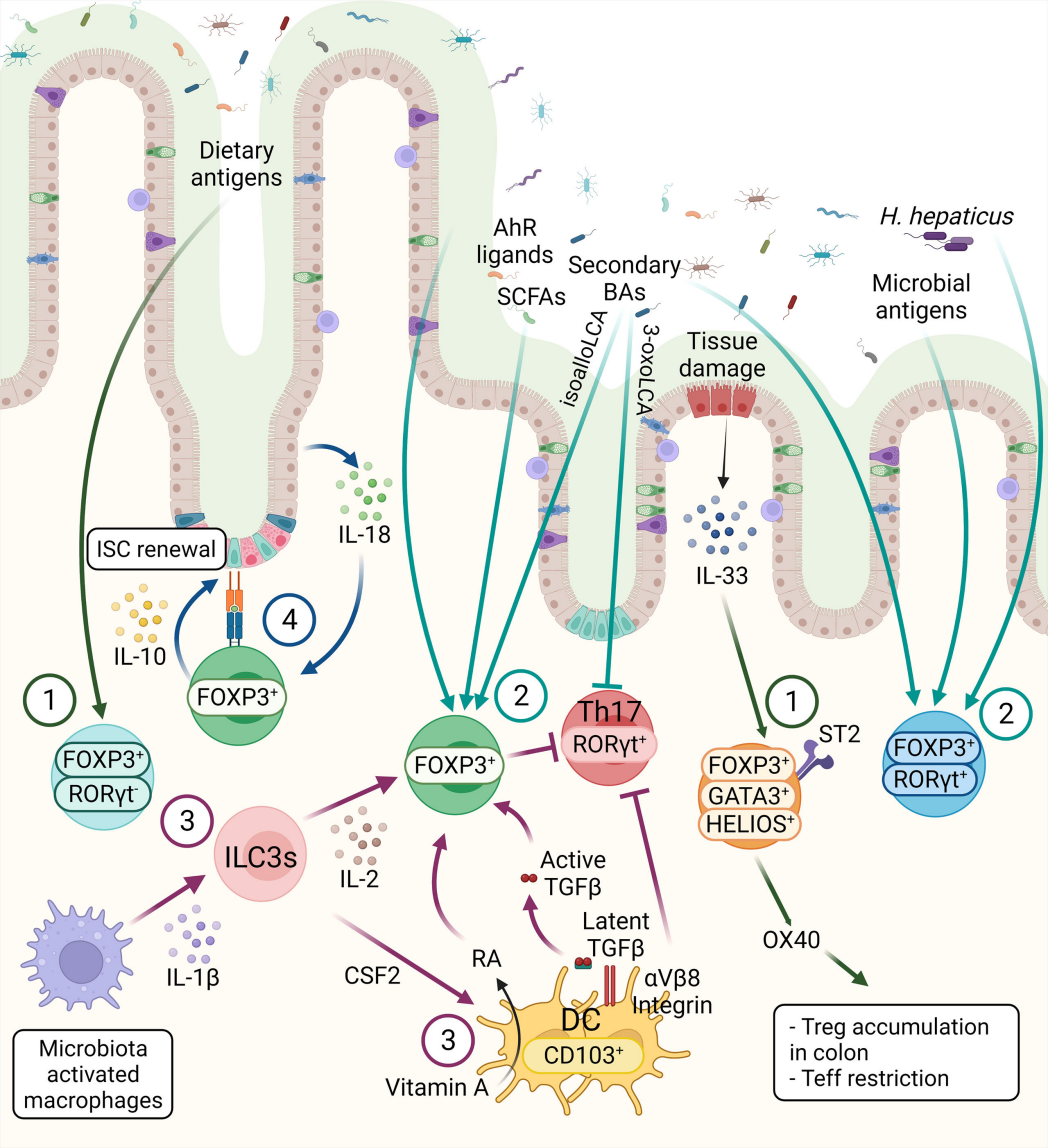
The three main subsets of FOXP3^+^ Tregs are controlled by host and environmental factors in the intestine. 1) The FOXP3^+^ RORγt^-^ Tregs are present mainly in the small intestine, mainly induced by dietary antigens. GATA3^+^ Tregs are thymic origin Tregs with high level of ST2, thus these cells can respond to IL-33, secreted from IECs in response to tissue damage. Moreover, OX40 expression on GATA3^+^ Tregs supports the accumulation of Tregs in the colon and OX40 is required for the Tregs-mediated restriction of effector T cells (Teff). 2) RORγt^+^ Tregs constitute the main colonic Tregs subset, promoted by the microbiota. SCFAs, secondary BAs and AhR ligands can induce Tregs differentiation, however the effect of these metabolites on different Tregs subsets are not known, except a recent finding showing that BAs induce RORγt^+^ Tregs. 3) CD103^+^ DCs induce the differentiation of Tregs in a TGF-β-dependent manner and suppress Th17 cells. These functions of DCs are regulated by αVβ8 integrin-mediated activation of latent TGF-β1 and RALDH-mediated metabolizing of vitamin A into RA. ILC3s, triggered by microbe-activated macrophages, promote Tregs in the intestine through IL-2 or CSF2 production. These microbiota induced Tregs, either through microbial metabolites or other immune cell types are essential for the maintenance of immune homeostasis. 4) The crosstalk between intestinal epithelium and Tregs are essential for intestinal homeostasis. Tregs can interact directly with ISCs *via* MHCII molecule and promote ISCs renewal by IL-10 signaling. IECs-derived IL-18 is an important regulator of Treg-mediated suppression of intestinal inflammation.

Tregs constitute more than 30% of the lamina propria (LP) CD4^+^ T cells in the colon and 20% of LP CD4^+^ T cells in the small intestine (28). Tregs, generated in the thymus, are characterized by the expression of IKAROS family zinc finger 2 (HELIOS) and Neuropilin-1 (NRP1). Interestingly, compared with Tregs in lymphoid tissues, HELIOS^+^NRP1^+^ Tregs constitute only 30% to 35% of the colonic Tregs in both mouse and human, suggesting peripherally derived Treg cells (pTregs) are the main population in the GI tract (27, 29–32). In small intestine, where is the primary site of nutritional absorption with abundant dietary antigens, the retinoic acid-related orphan receptor gamma-t (RORγt) negative pTregs are highly abundant that are responsible for maintaining immune tolerance against dietary antigens (**Figure 2**) (33). Around 50% of LP Tregs are dietary antigen specific RORγt^-^ pTregs, whereas only 15% of Tregs are RORγt^+^HELIOS^-^ Tregs that are primarily responsible for intestinal microbiota in small intestine (29). RORγt^+^ but not RORγt^-^ Tregs were diminished in antibiotics-treated mice, whereas the deprivation of dietary antigens led to severe reduction of NRP1^low^ pTregs due to the specific depletion of RORγt^–^ pTregs in the small intestine, suggesting while the microbial antigens are essential for the induction of RORγt^+^ pTregs, the induction of RORγt^-^ Tregs requires exposure to dietary antigens (33). Indeed, mice lacking NRP1^low^RORγt^–^ Tregs became more susceptible to food allergy (33).

On the other hand, the RORγt^+^ Tregs, which are highly abundant in the colonic LP, have unique roles to restrain intestinal inflammation, triggered by gut microbiota (34). In the colon, HELIOS^−^RORγt^+^FOXP3^+^ pTregs are the main source of Interleukin(IL)-10, which is necessary for maintaining intestinal homeostasis (**Figure 2**) (28). RORγt expression is essential for the pTregs to control commensal induced inflammation, while the Tregs-specific ablation of *Rorγt* causes hyper-production of the pro-inflammatory cytokines such as IL-17 and IFN-γ in FOXP3^-^ conventional CD4^+^ T cells (**Figure 2**) (29). The transcription factor c-MAF, encoded by musculoaponeurotic fibrosarcoma (*Maf*) gene, is important for maintaining the immune tolerance to bacteria by RORγt^+^FOXP3^+^ Tregs, supported by Tregs-specific deletion of *c-Maf* resulting in impaired IL-10 production and induction of bacteria-specific inflammatory T-helper 17 cells (Th17). This suggests a central role of c-MAF in the proper function of colonic RORγt^+^ Tregs (35). Moreover, Tregs-specific *c-Maf* deficiency has shown to trigger gut dysbiosis together with immune alteration with enhanced IgA-producing plasma cells and IL-17a/IL-22-producing Th17 in intestine indicating a key role of c-MAF in Tregs to maintain the homeostatic relationship between host and microbiota (36). In addition to the induction of tolerance against gut commensals, microbiota-dependent activation of the RORγt^+^ Tregs population is required for protection against food allergy (37).

A distinct colonic Tregs subpopulation is defined as the GATA-binding-protein (GATA)3^+^ HELIOS^+^ Tregs, which are mainly derived from thymus and play the key immunosuppressors during intestinal inflammation (**Figure 2**) (38). GATA3 expression is not necessary for the maintenance and proper function of Tregs at steady state, but essential for the accumulation of Tregs at inflamed sites during inflammation (39). Additionally, deletion of *Gata3* in Tregs results in spontaneous inflammation and intestinal pathologies with aging in mice (40, 41). Colonic GATA3^+^ HELIOS^+^ Tregs have been shown to express the IL-33 receptor ST2 (IL1RL1) which enable Tregs to expand in response to the alarmin IL-33 during inflammation (39). IL-33/ST2 engagement signals into Tregs to promote serine phosphorylation of GATA3 together with IL-2 and TCR engagement (**Figure 2**). This, in turn, increases the expression of *St2* and *Foxp3*, thus regulating the proliferation and maintenance of Tregs (**Figure 2**) (42). Furthermore, expression of OX40 by the GATA3^+^ST2^+^HELIOS^+^ Tregs subpopulation is essential for the accumulation of Tregs in the colon, as well as for the restriction of effector T cells in naïve T cell transfer model of colitis (**Figure 2**) (43).

Even though the colon harbors mainly pTregs, single-cell and high-throughput sequencing of the TCR repertoires of FOXP3^+^ Tregs revealed that the majority of the dominant TCRs are shared by colonic and thymic Tregs (44). This suggests that a vast majority of colonic Tregs might be of thymic origin, supported by the crucial roles of thymic Tregs (tTregs) to mediate the tolerance against intestinal antigens (44). A more recent study shows that early life colonization of bacteria in the intestine leads to the transport of microbial antigens from the intestine to the thymus by CX3CR1^+^ dendritic cells (DCs), hence promoting the development of microbiota-specific T lymphocytes (45). Further studies are needed to clarify whether microbiota-specific Tregs are induced in the thymus as well. Overall, these findings suggest that the colonic Tregs, which include both pTregs and tTregs, contribute together to maintain intestinal homeostasis.

## Microbial Regulation of Intestinal Tregs

The microbiota is crucial for the maturation of the immune system during early life (46). The “window of opportunity” theory suggests that the interaction between the microbiota and the host immune system during a critical developmental period might have long-lasting implications for disease susceptibility in later life (47, 48). During this time-window, goblet cell function as passages to facilitate the delivery of microbial antigens from the intestinal lumen to the CD103^+^ LP DCs for Tregs development (49). A transient depletion of the Tregs during weaning period (4 weeks of age) results in enhanced susceptibility to inflammatory pathology later in life (50), suggesting the unique roles of Tregs at early developmental stage by microbiota to maintain tissue homeostasis later in life.

Studies using germ free (GF) mice revealed the necessity of microbiota for intestinal Tregs homeostasis. GF mice present a decreased frequency of RORγt^+^ Tregs compared to specific-pathogen-free (SPF) mice which can be rescued by colonization with *Clostridium* species (51), altered Schaedler flora (ASF) (52), or *Bacteroides fragilis* (53). RORγt^+^FOXP3^+^ Tregs mediate the tolerance to a pathobiont, *Helicobacter hepaticus*, through restriction of pro-inflammatory Th17 in a transcription factor c-MAF-dependent manner (35). Several studies have revealed the regulatory role of the bacteria-derived metabolites, particularly short-chain fatty acids (SCFAs) (54–57) and bile acids (BAs) (58–60) in Tregs differentiation (**Figure 2**). SCFAs induce the differentiation of Tregs *via* T cell intrinsic upregulation of the *Foxp3* expression *via* either through inhibition of histone deacetylases (HDACs) at transcriptional level *via* G protein-coupled receptor (GPR) 43 (61), or by promoting histone acetylation in the conserved noncoding sequence region 1 (CNS1) of the *Foxp3* genomic locus (56). Moreover, SCFAs enhance GPR15 expression, which in turn induces Tregs accumulation in the colon (61, 62). However, a recent study proposed conflicting results, showing minor effects of dietary supplementation with SCFAs to promote pTregs induction neither in mesenteric lymph nodes nor the colonic LP, which might be explained by a ceiling effect related to the SCFA-producing microbiota frequency (63). Moreover, the bile acid metabolite, isoallolithocholic acid (isoalloLCA) promotes Tregs differentiation in a *Foxp3* CNS3-dependent manner through the production of mitochondrial reactive oxygen species (59). To the contrary, 3-oxolithocholic acid binds directly to RORγt and inhibits Th17 differentiation. One study revealed that isoalloLCA, produced by *Bacteroidetes* species in the gut, regulates the nuclear hormone receptor NR4A1 to promote the differentiation of naïve T cells to Tregs by enhancing *Foxp3* transcription (64). Similarly, the secondary bile acid, 3β-hydroxydeoxycholic acid, induces the generation of colonic RORγt^-^ Tregs *via* a *Foxp3* CNS1-dependent manner in DCs-intrinsic the farnesoid X receptor activity suggesting an interaction between bile acid and nuclear receptor (58). Furthermore, colonizing GF mice with *Bacteroides* species promotes RORγt^+^ Tregs in the colon *via* the vitamin D receptor (VDR) in a BAs-dependent manner (60). While the mice fed minimal diet developed severe dextran sodium sulfate-induced colitis, the BA supplementation increased the numbers of RORγt^+^ Tregs and alleviated the disease. Moreover, Tregs-specific VDR deficiency worsened the DSS-induced colitis. Overall suggesting both BAs and VDR have a protective role in chemical-induced colonic inflammation by modulating RORγt^+^ Tregs (60). Finally, tryptophan metabolites are important components of intestinal immune tolerance by Tregs *via* aryl hydrocarbon receptor (AhR) (**Figure 2**) (65). Intestinal Tregs express *Ahr* at higher levels compared to Tregs in the spleen or lymph nodes (66). In a T cell transfer model of colitis, the suppressive effect of Tregs on intestinal inflammation was diminished by the specific depletion of *Ahr* in Tregs (66). Whole-body *Ahr* deficiency acutely attenuates the expression of GPR15 both in effector memory T cells and in Tregs (67). Like SCFAs, AhR upregulates GPR15 expression together with FOXP3 in Tregs. Conversely, RORγt acts as an antagonist of AhR DNA binding to the *Gpr15* locus in T lymphocytes to suppress *Gpr15* transcription (68). Overall, these findings highlight the role of the microbial metabolites in controlling host immune responses by acting on intestinal Tregs.

## Crosstalk Between Intestinal Tregs and Other Immune Populations

Tregs communicate with various immune cell types in a cooperative way to maintain immune tolerance. In the gut, antigen presenting cells (APCs) interact with conventional CD4^+^ T cells and promote the development of pTregs (**Figure 2**). Intestinal APCs, expressing the chemokine receptor CX3CR1, induce Tregs differentiation while limiting T effector cell expansion against soluble antigens and the microbiota itself in IL-10 dependent manner (**Figure 2**) (69). CD103^+^ DCs of the intestinal LP can similarly present luminal antigens by capturing them to extend their dendrites to the lumen through the intestinal epithelium (**Figure 2**) (70). DCs have control over the TGF-β-dependent differentiation of naïve T cells, promoting Tregs but suppressing Th17 *via* integrin αVβ8-mediated activation of latent TGF-β1 and retinal dehydrogenase (RALDH)-mediated metabolizing of vitamin A into retinoic acid (RA) (**Figure 2**) (71–74). Interestingly, stromal cells in the intestinal LP can upregulate RA from DCs in an RA- and granulocyte-macrophage colony-stimulating factor-dependent manner. In turn, stromal cell-primed DCs enable to induce Tregs (**Figure 2**) (75). RORγt^+^ type 3 innate lymphoid cells (ILC3s) are another immune cell type in the intestine that regulates FOXP3^+^ Tregs differentiation (**Figure 2**). The crosstalk between IL-1β-secreting macrophages and Colony stimulating factor 2 (CSF2)-producing RORγt^+^ ILC3s has been proposed a mechanism of Tregs regulation by ILC3s in the intestinal mucosa. The gut microbiota induces IL-1β production by macrophages to enhance colonic Tregs frequency by upregulating ILC3s-derived CSF2 (**Figure 2**) (76). IL-2, produced by ILC3s as a response to the APC-derived IL-1β upon microbial stimulation, is essential for Tregs maintenance and immune tolerance. In addition, IL-2-producing ILC3s are important for the oral tolerance of dietary antigens in the small intestine, while the decrease of IL-2 production from ILC3s is associated with lower Tregs frequencies in Crohn’s disease (**Figure 2**) (77). Reciprocally, both Tregs and Th17 have control over ILC3s in the intestine (**Figure 2**) (78). In the absence of CD4^+^ T cells, ILC3s displayed a comprehensive and persistent phosphorylation of Signal transducer and activator of transcription 3 (STAT3), as an outcome of hyper-production of IL-22 by ILC3s in response to microbiota (78). Adoptive T cell transfer experiments revealed that Tregs can inhibit ILC3s activation by reducing IL-23, produced by CCR2^+^ monocytes and monocyte-derived DCs, whereas Th17 lower the bacterial burden, thus limiting ILC3s activation (78). In agreement with these results, another study using anti-CD40-driven colitis model showed that Tregs exert a protective function by reducing IL-22 secretion from ILC3s *via* the suppression of production of IL-23 and IL-1β in CX3CR1^+^ macrophage by latent activation gene 3 (LAG3)-major histocompatibility complex class II (MHCII) engagement (79).

## Interactions Between Intestinal Tregs and Intestinal Epithelial Cells

Communication between the immune and non-immune cell populations has recently drawn considerable attention in intestinal health and disease (80–83). The intestinal epithelium forms a barrier between microbes and host to coordinate the crosstalk between the gut microbiota and the mucosal immune cells, while epithelial cells also respond to immune and microbial stimuli (84). Tregs maintain the epithelial barrier integrity in the intestine by promoting intestinal stem cell (ISC) renewal *via* IL-10, whereas effector T cell subsets induce ISC differentiation, as demonstrated by organoid studies (**Figure 2**) (83). Accordingly, *in vivo* Tregs deficiency results in a reduced ratio of ISCs to differentiated intestinal epithelial cells (IECs) (83). By facilitating the direct interaction between IECs and T cells, the expression of MHCII on intestinal epithelial cells likely contributes to T cell cytokine-mediated ISC renewal and differentiation. A lack of MHCII in IECs reduced the levels of surface MHCII on the intestinal mononuclear phagocytes and the proportion of HELIOS^–^ microbial-responsive Tregs in the small intestine, suggesting a communication network between the mononuclear phagocytes, IECs, and Tregs (85). IECs-specific MHCII deficiency led a slight reduction of Tregs frequency in the colon LP during steady state (86). A recent study reported that mice lacking IECs-intrinsic MHCII have expanded commensal-specific cBir1^+^ CD4^+^ T cells, specifically recognizing bacterial flagellin. In a fungal commensal model, mice colonized with *Candida albicans*-2W1S^+^ displayed increased numbers of *C. albicans*-specific 2W1S^+^ CD4^+^ T cells in the large intestine upon IEC-specific MHCII deficiency (87). Deletion of IEC-intrinsic MHCII expression altered the ratio of Tregs to Th17 in both commensal-specific cBir1^+^CD4^+^ T cell and C. albicans-2W1S^+^ commensal models (87). Collectively, these studies indicate that the disruption of epithelial MHCII-T cell interaction modulates microbiota-specific immune responses in the intestine.

Tregs-IECs interactions occur not only *via* MHCII but also through cytokine signaling (88). In the colon, IECs participate in the regulation of CD4^+^ T cell homeostasis *via* IL-18 production (**Figure 2**) (88). In the homeostatic condition, IECs-derived IL-18 is dispensable for Tregs differentiation, but Tregs-mediated suppression of intestinal inflammation requires IL-18/IL-18R1 signaling in the T cell-transfer colitis model (88). Another member of the IL-1 family, IL-33, is constitutively expressed in epithelial cells, including IECs (89). In the inflamed colon, epithelial IL-33 levels are elevated (42). After being released from IECs upon tissue damage, IL-33 promotes Tregs function and adaptation to the inflammatory environment through ST2 signaling (42). IL-23 inhibits Tregs responsiveness to IL-33, implying that the balance between IL-33 and IL-23 might be a key regulator of intestinal Tregs homeostasis (42). The clear evidence indicates a reciprocal interaction between IECs and intestinal Tregs for intestinal homeostasis; nevertheless, there is still much to learn about the crosstalk of Tregs with IECs, as well as other non-immune populations in the intestine.

## Tregs in the Central Nervous System

Previously, the CNS was considered an immune-privileged site due to a lack of lymphatic vasculature and the presence of the blood-brain barrier (BBB) (90). This view led to undervalue the roles of immune system in CNS; however, it has been revisited in light of the recent identification of functional lymphatic vessels (91) and immune cells including T cells in the meninges (90). Furthermore, T cells have found in the brain parenchyma of healthy mice, though small numbers (about ~2,000 CD4^+^ T cells and ~150 Tregs in the entire brain) (92). Post-mortem human studies also identified T cells in the pathological (93) and healthy (92) brain. T cells are actively involved in the pathologies of CNS disorders and injuries by infiltrating into the CNS (94). Though often neglected due to their scarcity, recent studies have enlightened the essential roles of T cells in CNS physiologies. For instance, meningeal T cell-derived IFN-γ regulates neuronal connectivity, promoting an inhibitory current in cortical gamma aminobutyric acid (GABA)ergic neurons (95). A recent study showed that brain-resident CD4^+^ T cells are required for the maturation of microglia (92). The existence of Tregs in the CNS implies their potential roles in CNS homeostasis (92). Here, we discuss the characteristics of CNS-resident Tregs and provide an overview of how Tregs interact with other CNS cells, such as neurons and glial cells.

## Origin and Entry of Tregs Into the Central Nervous System

Blood-derived T cells can migrate into the CNS through at least three major routes: to the perivascular space through the BBB, through the subarachnoid space in the meninges, and to the cerebrospinal fluid (CSF) across the choroid plexus (96). However, T cell entry into the brain parenchyma is limited by the glia limitans between the perivascular space and parenchyma, and by epidermal cells between the CSF space and parenchyma (97). In inflammatory conditions, the number of infiltrating T cells, including Tregs, increases due to exacerbated permeability or disruption of the BBB and the blood-CSF barrier (98). In addition, the altered chemokines niche actively participate to recruit T cells in the insulted brain region (97). In particular, in the middle cerebral artery occlusion (MCAO) model of stroke, the infiltration of Tregs into the infarcted brain region is driven by chemokines such as CCL1 and CCL20 (22).

Like other tissue-resident Tregs, CNS Tregs require TCR recognition specific to antigen in the CNS, in both physiological (92) and pathological conditions (22). Studies using OT-II transgenic mice, which express ovalbumin-specific TCR, demonstrated no OT-II Tregs in the brains of these mice (22, 92). Furthermore, in the experimental autoimmune encephalomyelitis (EAE) model, Tregs in the CNS showed over-representation of specific Vβ8 TCR, suggesting oligoclonal expansion of Tregs against self (or potentially brain specific) antigens in CNS (99). This is different from conventional CD4^+^ T cells in CNS, which require peripheral activation (92). In agreement with this, TCR sequencing of Tregs in brain revealed several overlapping TCR clones, especially TCRα across individual mice, which suggests the shared antigens in CNS Tregs (22). Tregs can proliferate inside the CNS as well. For instance, Tregs in CNS can actively proliferate in the EAE model (99), and the amplification of brain Tregs is dependent on cytokines such as IL-2 and IL-33 in the MCAO model (22, 100). Interestingly, the neurotransmitter serotonin also exerts a proliferative effect on brain Tregs through a serotonin receptor signaling (22).

From the perspective of the gut-brain axis, it has been proposed that the gut microbiome can affect the homing of T cells, including Tregs, into the CNS. When SPF mice were co-housed with dirty pet shop mice, CD4^+^ T cells but not Tregs significantly increased in brain (92). One study with T cells expressing a photoconversion fluorescent protein showed the migration of intestinal T cells to the cervical lymph nodes and meninges after ischemic stroke (101), implying a role for gut-resident T cells in CNS pathogenesis.

One possible hypothesis is that gut microbiota-derived molecules may act as antigens in brain, which are necessary for Tregs trafficking to the CNS (22, 92). The ‘molecular mimicry’ hypothesis has been investigated in various nervous autoimmune disease models, such as EAE (102) and a model for Guillain–Barré syndrome (GBS) (103). The mono-colonization of *Lactobacillus reuteri*, which possesses peptides that potentially mimic myelin oligodendrocyte glycoprotein (MOG), exaggerated EAE symptoms than those of GF (102). The structure of lipooligosaccharide (LOS) on *Campylobacter jejuni* is similar to Ganglioside GM1, and GM1-like LOS sensitized rabbits show pathology similar to GBS such as flaccid paralysis (103). Furthermore, an adenosine triphosphate-binding cassette transporter of *Clostridium perfringens* shared sequence homology with Aquaporin-4 (AQP4) (104), of which autoantibodies are pathogenic for neuromyelitis optica spectrum disorders (NMOSD) (105). Indeed, strains of *C. perfringens* are abundant in patients with NMOSD (104). Further studies are required to address the involvement of the gut microbiome in the actions of CNS-resident Tregs.

## Characteristics of CNS-Resident Tregs

Due to the small number of CNS-resident Tregs in homeostatic conditions, their characteristics are not well investigated. Brain-resident Tregs are highly distinct from blood Tregs in adult mice, with elevated expression of activation markers including CD44, CTLA4, and ICOS, and expression of residency markers such as ST2 and CD69 (92). Parabiosis of *Foxp^Thy1.1^Cd45.1* mice with *Foxp3^Thy1.1^Cd45.2* mice showed the presence of CD69^+^ Tregs in host brain tissue after approximately 7 weeks, in contrast to the rapid exchange of blood Tregs (92).

**Figure 3:**
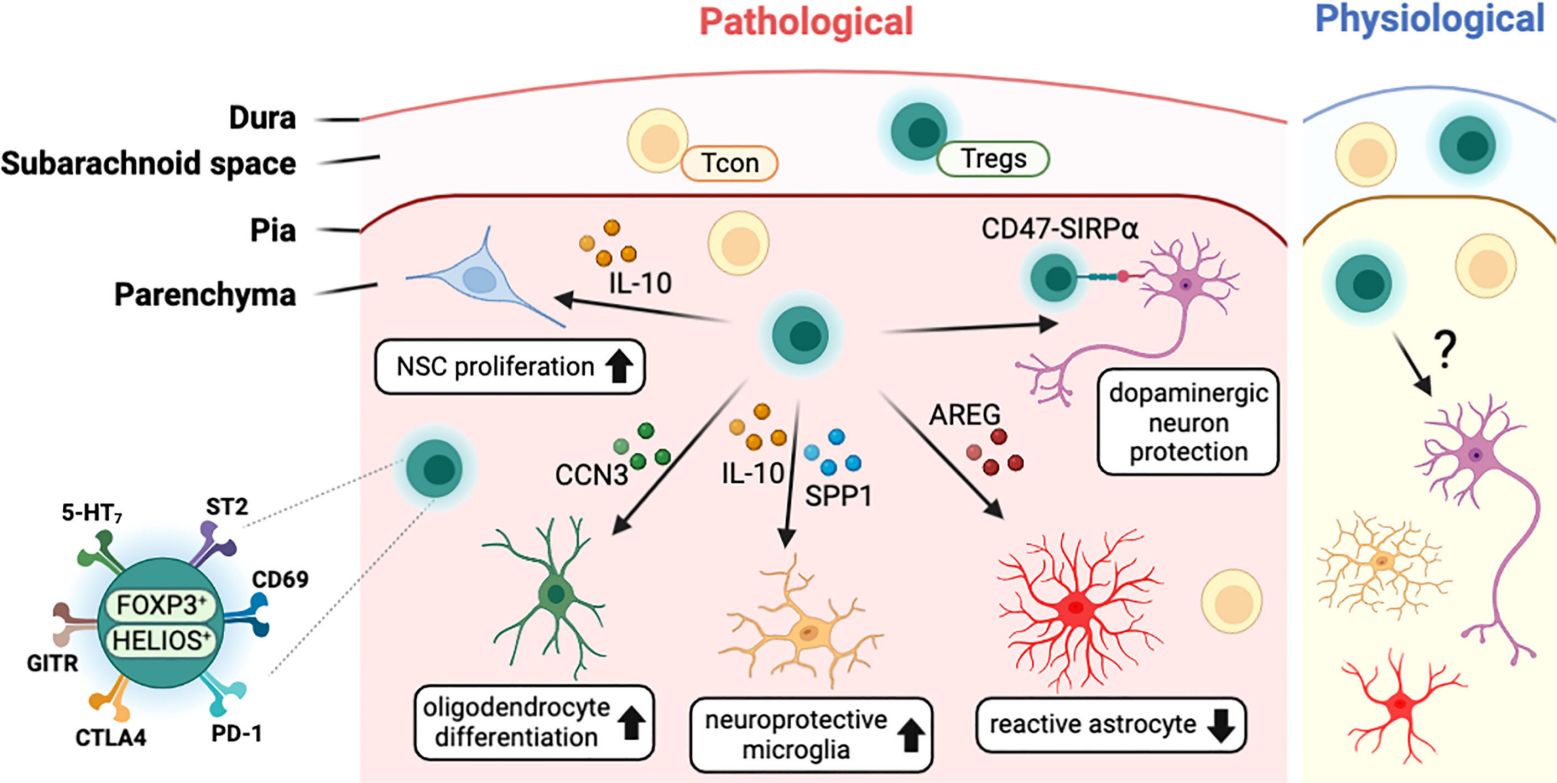
Characteristics of CNS-resident Tregs and their interaction with other CNS cells. T cells including Tregs exist in the meningeal space and the brain parenchyma in both physiological and pathological conditions. In disease status, Tregs interact with CNS cells such as neural stem cells (NSCs), oligodendrocytes, microglia, astrocytes, and neurons to modulate the pathology of CNS insults. CNS-resident Tregs express a high level of Treg markers such PD-1 along with neuronal receptors including 5-HT7. CCN3, cellular communication network factor 3; SPP1, osteopontin; AREG, amphiregulin; SIRPα, signal regulatory protein alpha; 5-HT7, serotonin receptor 7; ST2, interleukin-1 receptor-like 1; PD-1, programmed cell death 1; CTLA4, cytotoxic T-lymphocyte-associated protein 4; GITR, glucocorticoid-induced TNFR-related protein.

Several studies with stroke models have found that T cells, including Tregs, infiltrated into the infarcted brain region (**Figure 3**). In the healthy brain, the infiltrated Tregs showed activated phenotypes (CD44^hi^/CD62L^lo^) and expressed high levels of canonical Tregs markers such as PD-1, CTLA4, GITR, and CD103 (22, 106). Transcriptome analysis of brain-infiltrated Tregs showed a profile similar to those of other tissue Tregs such as visceral adipose tissue and muscle (107, 108), with the expression of *Il-10*, amphiregulin (*Areg*), *Klrg1*, *Pparγ*, and WNT signaling-related genes (22). In both healthy and pathological conditions, CNS-resident Tregs are mostly positive for HELIOS suggesting thymic origin (22, 92, 106). In addition, CNS-resident Tregs may acquire CNS-specific phenotypes, as do other tissue-resident Tregs. Interestingly, after stroke, brain-resident Tregs express several neuropeptides such as neuropeptide Y (*Npy*) and preproenkephalin (*Penk*), and neuronal receptors including serotonin receptor type 7 (*Htr7*) and arginine vasopressin receptor (*Avpr1a*) (22). This indicates potential interaction between Tregs and other cells in the nervous system. Comparison between blood and brain infiltrating Tregs show that various cytokines (*Spp1*, *Il10*) and trophic factors (*Igf1*, *Osm*) are also increased in brain Tregs (106). However, as most of these studies were performed in pathological conditions, determining whether these unique characteristics of CNS-resident Tregs can be generalized to brain homeostasis in health and disease will require further investigation. Moreover, the clarification of environmental signals and cellular mechanisms used by Tregs for CNS homeostasis may be essential to understand the role of CNS-resident Tregs.

## Tregs Interact With Central Nervous System-Resident Cells

In general, immunomodulatory functions of Tregs are exerted by interacting with other immune cells. The unique characteristics of CNS-resident Tregs imply to regulate various CNS processes by interacting with CNS-resident cells *via* distinctive mechanisms other than classical Tregs functions. In this section, we will discuss how CNS-resident Tregs interact with each cell type and how they can regulate CNS function in heath and disease (**Figure 3**).

### Astrocyte

Astrocytes are one type of glial cells that regulate a variety of physiological properties, such as the production of neurotrophic factors and the regulation of neuronal development and neuronal synapses (109, 110). In pathological conditions, astrocytes become reactive and often neurotoxic (111). The neuroprotective role of brain Tregs has been studied by repressing neurotoxic astrogliosis. In the context of traumatic brain injury, peripheral T cells are infiltrated into the brain to trigger astrogliosis that is exacerbated in diphtheria toxin-inducing Tregs-depleted mice (112).

In the stroke mouse model, glial fibrillary acidic protein (GFAP)^+^ astrocytes and CD4^+^ T cells, especially Tregs, are accumulated at the ischemic injury site at 14 days after MCAO, and this stockpile of astrocytes significantly increased upon Tregs-depletion (22). AREG was abundantly produced by brain Tregs than splenic Tregs, which are known for wound healing and tissue repair (113). Astrogliosis in the brain of MCAO mice was diminished by transferring wild-type (WT) Tregs but not *Areg*-deficient (*Areg* ^-/-^) Tregs (22). Furthermore, IL-6 expression by microglia and astrocytes was reduced in WT but not *Areg* ^-/-^ Tregs-transferred mice. This indicates that brain Tregs produce AREG to suppress the neurotoxic astrogliosis by suppressing IL-6 production from astrocytes (22). IL-33-ST2 signaling played the key roles for Tregs infiltration into the brain that result in increased expression of AREG and epidermal growth factor receptor (EGFR) at MCAO (100). As neurotoxic astrogliosis is harmful in many other CNS injuries (111), it would be interesting to determine whether the Tregs-mediated strategy is applicable in other CNS diseases. Astrocyte-producing molecules can affect CNS resident Tregs vice versa. Co-culturing astrocytes with splenic T cells showed that astrocytes help to sustain FOXP3 expression in Tregs through IL-2/STAT5 signaling (114). Recently, a study reported higher circulating Tregs and serum IL-10 level at 48 and 72 hours after stroke onset in patients (115). To further identify the association of Tregs frequency with clinical outcomes, stroke patients are divided into two groups based on disease outcome (good vs. poor based on modified Rankin scale) and found the poor clinical outcomes with a higher infection risk especially in patients with lower Tregs frequency at 48 hours after stroke (115).

Despite the lack of clear mechanisms for the interaction between Tregs and astrocytes in brain, these studies imply therapeutic potential of Tregs to control astrocytes that should be further investigated in future.

### Microglia

Microglia are resident macrophages in the brain, which compose about 6% to 18% of the human brain neocortical cells (116, 117). Not like other CNS cells, microglia are originated from the yolk sac during the embryonic period (118) and maintain brain homeostasis and neuronal development *via* various cytokine signaling (119, 120). In the brain, IL-10 from T cells and natural killer cells (NKs) prevents deleterious microglial hyperactivation following peripheral endotoxin challenge (121). Likewise, IL-10 from Tregs modulates the alternative (M2) microglial polarization to ameliorate the outcome of intracerebral hemorrhage (122). In addition, FOXP3^+^ Tregs are expanded under co–cultured condition with MHCII^+^CD40^dim^CD86^dim^IL-10^+^ microglia, stimulated by low dose IFN-γ/MOG, resulting in mitigating the EAE severity (123).

Microglia are involved along with astrocytes in the context of inflammation. In murine stroke model, brain Tregs secrete osteopontin (SPP1) to promote tissue-regenerative microglial reactions for brain repair through the Integrin beta-1 (ITGB1) receptor, expressed on microglia (106). Cerebral Tregs secrete higher level of IL-10 than splenic Tregs, which has the key role to control the LPS-induced inflammation in microglia (114). Furthermore, co-culture of Tregs with microglia, promotes the expression of various factors linked with brain repair and anti-inflammatory processes in microglia (106).

In neurodegenerative diseases, Tregs have shown their potential to delay disease progress by modulating microglial function. Amyotrophic lateral sclerosis (ALS), which is characterized by the selective destruction of motor neurons, involves lymphocyte infiltration into the CNS and activation of microglia in mice and human (124, 125). In the superoxide dismutase 1 (SOD1) transgenic mouse, which is a murine model for ALS, Tregs suppressed the cytotoxic microglial factors such as NOX2 and iNOS in IL-4 dependent mechanism (126). Indeed, compared with healthy individual, Tregs from ALS patients express lower level of *Foxp3* mRNA together with the impaired suppressive function that are positively correlated with progressive rate and severity of ALS disease (124).

In an AD mouse model (APP/PS1), the depletion of Tregs exacerbated cognitive dysfunction, accompanied by reducing the recruitment of microglia toward the amyloid beta plaques and lingered disease-related gene expression profile, and behavioral impairments, which were rescued by enhancing Tregs with low-dose IL-2 treatment (127). Indeed, circulating Tregs were significantly reduced in patients with mild cognitive impairment (128). Moreover, in a Parkinson’s disease (PD) mouse model, neurotoxic microglial activation was ameliorated by adoptive transfer of Tregs (129).

The significance of Treg-microglia interaction in conditions other than CNS damage or neurodegenerative illness is an interesting subject. Severe neuroinflammation is often observed in the brain with schizophrenia (130) or stress-induced depressive disorder (131, 132) in human and mice (133). Given that psychosis is accompanied with inflammatory responses in microglia, it is hypothesized that Tregs may contribute to the regulation of the microglial-induced inflammatory responses in mental disorders.

### Oligodendrocyte

Oligodendrocytes are myelinating glial cells that support neuronal signals and produce the insulating sheath covering axons in the CNS (134). If remyelination fails, the damaged myelin leads to irreversible axonal loss and demyelinating diseases like multiple sclerosis. In a lysolecithin induced demyelinated mouse model, Tregs promoted oligodendrocyte differentiation and remyelination by producing communication network factor 3 (CCN3), a growth regulatory protein (21). On the other hand, Th17 have shown to inhibit oligodendrocyte maturation and survival through IL-17 (135). Further studies are necessary to understand the intercommunication between Tregs and oligodendrocyte for brain homeostasis.

### Neuron

There is limited evidence that Tregs directly affect neurons; however, in an 1-methyl-4-phenyl-1,2,3,6- tetrahydropyridine (MPTP) induced-PD mouse model, adoptive transfer of Tregs attenuated behavioral change, the inflammatory reaction in the brain, and the loss of tyrosine hydroxylase-positive dopaminergic neurons in the substantia nigra (136), partially due to direct interaction between Tregs and dopaminergic neurons *via* CD47 and SIRPα, respectively (137). On the other hand, neurological molecules can govern the response of CNS-infiltrated Tregs (133). Neurons, co-cultured with T cells, produced TGF-β1 and B7 molecule, to convert encephalitogenic T cells to TGF-β1^+^CTLA4^+^ Tregs which potentially inhibit disease progression upon transferring into EAE model mice (138).

### Neural Stem Cells

Following an insult to the CNS, *de novo* neurogenesis to replace the damaged neuron is important for functional recovery (139). Neural stem cells (NSCs), localized in specific regions of the adult brain, such as the subventricular zone and the dentate gyrus of the hippocampus, can replenish new neurons (140). Considering the unique roles of Tregs for the regulation of stem cells in skin (141), it is plausible that Tregs participate the regulation of neural stem cells in brain. Indeed, depletion of Tregs *via* anti-CD25 treatment led to impaired neurogenesis after stroke in a mouse model (142). Moreover, the transferring of activated Tregs enhanced NSCs proliferation in the subventricular zone (SVZ), which was mediated by IL-10 produced by Tregs (143).

Given the role of Tregs in pathological conditions, further study is necessary to determine whether Tregs interact with NSCs for neurodevelopment or homeostatic adult neurogenesis. In mice, lymphocytes are found in the brain during the perinatal period (92, 144), implying their role in brain at developmental stages. It is worth mentioning that a subset of B cells, B-1a cells, are involved in oligodendrogenesis during brain development (144). The EGFR ligand AREG, which is mainly produced by tissue Tregs, might be one candidate for regulating NSCs by Tregs in brain, as EGFR signaling is important for NSCs to maintain their proliferative capacity during cerebral cortex development (145). Furthermore, AREG acts as the mitogen for adult NSCs (146).

## Crosstalk Between Intestinal Tregs and the Nervous System

**Figure 4:**
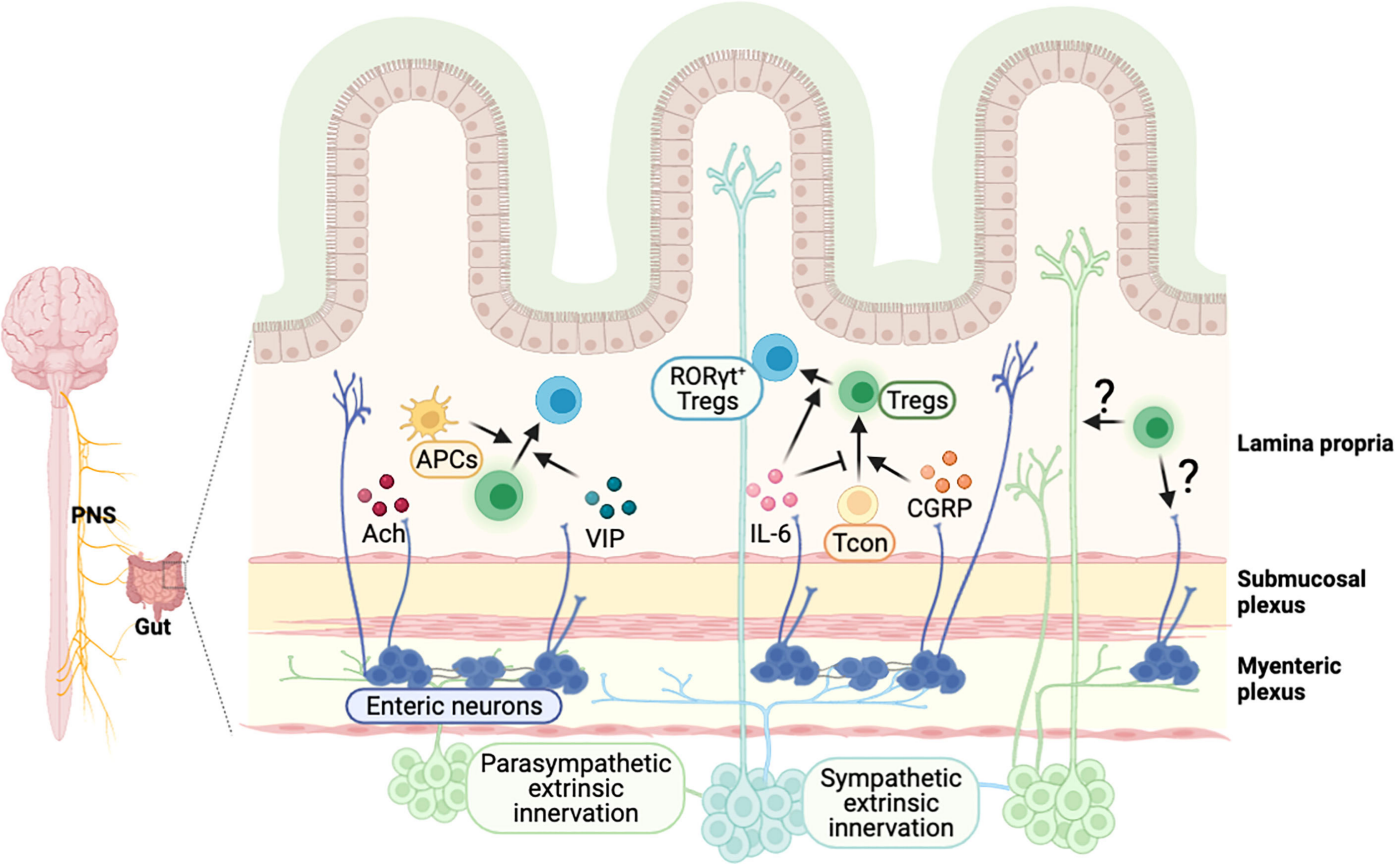
Possible model of crosstalk between intestinal Tregs and gut-innervating neurons. External neuronal cells from the peripheral nervous system innervate to the intestine, and neurons in the enteric nervous system exist in the colonic myenteric and submucosal plexus. Neuronal factors, released from the nerve terminal, are involved in the regulation of intestinal Tregs. A neurotransmitter acetylcholine (Ach) activates colonic antigen presenting cells, which may support the development of pTregs in the intestine. Vasoactive intestinal peptide (VIP) enhances the proliferation of Tregs and calcitonin gene-related peptide (CGRP) promotes the differentiation of Tregs. Cytokines such as IL-6, produced by neuronal cells, regulate the induction of RORγt^+^ Tregs. It is expected that Tregs-derived molecules can signal to neuronal cells to modulate their function, which have to be further elucidated.

Neurons in the PNS innervate various organs in the body. It has been implied that neuronal signaling can regulate the immune system in other organs, as immune cells are known to express several receptors for neurotransmitters and neuropeptides (11, 65, 147). Consistently, a line of human studies shows the dysregulation of the immune system in neurological diseases. In particular, the imbalance of Th17 and Tregs in the peripheral immune system have been reported in patients with autism spectrum disorder (148, 149), epilepsy (150), PD (151), and schizophrenia (152). The gut and brain communicate with each other, and their close association implies reciprocal control between the nervous system and intestinal immunity. Focusing on Tregs, we cover the new discoveries and perspectives on the regulation of intestine Tregs by neuronal signals and vice versa (**Figure 4**).

## Neuronal Signaling Regulates Intestinal Tregs

The GI tract is innervated by various peripheral neuronal cells – sympathetic, parasympathetic, and sensory neurons (153) – that regulate various physiological functions. PNS neurons can directly innervate into the GI tract or deliver neuronal signals through the ganglia, which are a collection of neuronal cell bodies in the periphery (153–155). Efferent sympathetic and parasympathetic neurons convey signals from the brain to the gut. Sympathetic neurons are originated from the spinal cord and project to the prevertebral ganglia and pelvic ganglia. Parasympathetic neuron cell bodies are located in the dorsal motor nucleus of the vagus nerve in the hindbrain or in the lumbosacral spinal cord, and they project to the pelvic ganglia or directly to the GI tract (154, 155). Afferent sensory neurons originating from nodose/jugular ganglia and dorsal root ganglia are pseudo-unipolar neurons that extend into both the peripheral organs such as the gut and the CNS. Afferent neurons convey information from the gut to the brain (153), but also can signal to the gut by releasing neuropeptides (156). Besides the external innervations, the GI tract has an independent nervous system known as the enteric nervous system (ENS), connected with external neurons conveying signals from the CNS (157).

Although the exact mechanism is not well understood, neuronal signals can regulate intestinal Tregs (**Figure 4**). Indeed, vagus nerve stimulation increased frequency of intestinal Tregs (158), but vagotomy reduced the number of Tregs in the colon, particularly HELIOS^−^RORγt^+^ pTregs (11). Neurotransmitters and neuropeptides, released from the nerve terminal, have been proposed as the potential mechanisms for the regulation of T cell plasticity in the GI tract. Cholinergic parasympathetic neurons produce acetylcholine (159). In addition, nicotinic and muscarinic classes of acetylcholine receptor expressed on T cells regulate T cell differentiation *in vitro* (160). Vasoactive intestinal peptide (VIP) is a neuropeptide, known for its anti-inflammatory function, produced by neurons in PNS (161) and ENS neurons (162). VIP induces proliferation of CD4^+^CD25^+^ Tregs producing IL-10 and TGF-β in lymph node and spleen (163), which has potent therapeutic effect in inflammatory disorders including EAE (164) and collagen-induced arthritis (165). Calcitonin gene-related peptide (CGRP), a pain-related neuropeptide, mainly released from sensory neurons (166), increases differentiation of Tregs in a model of EAE (167). On the other hand, substance P, a mediator for pain neurotransmission secreted at the end of sensory neuron nerve (168), was shown to impair Tregs function in murine dry eye disease model through the neurokinin 1 receptor, expressed on Tregs (169). Other signaling molecules, such as cytokines, produced by neuronal cells, may also regulate intestinal Tregs. For example, ENS neuron-derived IL-6 regulates RORγt^+^ Tregs differentiation (170) and epithelial-derived IL-18 regulates Tregs function in a colitis model (88). Furthermore, high level of cytokine, chemokine, and their receptors are detected in ENS neurons (171, 172). Overall, these findings suggest various neuron-derived messenger molecules are involved in the regulation of intestinal Tregs and homeostasis.

Besides signaling through the receptors expressed on Tregs, neuronal signaling can indirectly regulate intestinal Tregs *via* other intestinal immune cells. For instance, the neurotransmitter acetylcholine, produced by enteric neurons, activates muscarinic acetylcholine receptors (mAchR) on colonic APCs, such as CX3CR1^+^ mononuclear phagocytes and CD103^+^ DCs to promote pTregs differentiation *via* aldehyde dehydrogenases (11).

As discussed, not only neuronal signaling would enables to modulate intestinal Tregs in various pathological conditions, but also local tissue Tregs may modulate the function of peripheral neurons. This has been studied in murine pain model such as chronic constriction injury, in which spontaneous pain recovery after the injury was delayed in Tregs-depleted mice (173, 174). While these studies do not give direct evidence of Tregs on peripheral nerves, this is enough to suggest the essential roles of inflammation for the repair and function of an innervated nerve.

## Microbiome and Intestinal Treg in the Gut-Brain Axis

As discussed, the microbiome is an important regulator of immune system. Several studies have shown that the gut microbiome can regulate neurons innervating the GI tract, which, in turn, may lead to the modulation of intestinal Tregs. This entanglement potentially influences CNS activity and behavior *via* signaling through the gut-brain axis. For example, GF mice show more anxiolytic and anti-social behavior pattern than SPF mice (175). In addition, recent studies indicate that the microbiome regulates transcriptome and neural activity of organ-innervated neuron. The activity of gut-innervated sympathetic neurons is increased in GF or antibiotics-treated mice as shown by staining with c-FOS, indicating direct regulation of neuronal activity by commensal microbiota (13). In RNA-sequencing based transcriptome analysis with myenteric neurons from GF and SPF mice, *Ahr* expression is significantly increased depending on the microbiome that regulates intestinal peristalsis (176). Moreover, the gut microbiota induced neuronal maturation in the colonic myenteric plexus of GF mice, colonized with a normal microbiota, *via* serotonin type 4 (5-HT4) receptor signaling (177). Regulation of neurons, involved in the gut-brain axis by the gut microbiome, plausibly modulates the immune composition and function in the GI tract, including Tregs. An intestinal organ culture system showed that Tregs-inducing *Clostridium ramosum (C.ramosom)* altered neurotransmitter expression in gut, which regulated RORγt^+^ Tregs (178). In the reverse, gut-innervating nociceptor neurons not only produced CGRP but also shaped the composition of gut microbiota such as segmented filamentous bacteria (SFB) to promote host defense against Salmonella in the small intestine (156). Altogether, these studies suggest the essential roles of gut microbiome for the proper regulation of gut-brain axis *via* Tregs.

## Conclusion and Perspective

In recent years, emerging studies have shown the role of Tregs in regulating pathophysiological condition along the gut-brain axis. Given the functional diversity and heterogeneity of gut-resident Tregs as described in this article, it is well-demonstrated that intestinal Tregs are crucial for the maintenance of immune homeostasis and tolerance to luminal antigens along with pathogens. In addition, CNS-resident Tregs, which interact with various CNS cells including neurons, glial cells, neural stem cells, etc. govern brain homeostasis (**Table 1**). Since present knowledge about CNS-resident Tregs is limited in pathological or inflammatory conditions in which most of CNS T cells are actively infiltered from peripheral immune system, it remains to be clarified whether Tregs in the CNS, despite their limited number, have physiological roles in the homeostatic state.

**Table 1:** Interaction of Tregs with tissue-resident cells and its physiological outcomes in the CNS and the intestine.

Region	Target cell	Physiological outcome	Reference
CNS	Astrocyte	Diminishment of astrogliosis in MCAO stroke mouse model by transferring wild-type Tregs *via* AREG signaling on Tregs	(22)
		Strengthened *Foxp3* expression in Tregs through IL-2/STAT5 signaling by *in vitro* co-culturing with astrocytes	(114)
	Microglia	Inhibition of inflammatory response (TNFα, IL-6, IFN-γ) of LPS-stimulated microglia and pathology of intracerebral hemorrhage *via* IL-10 signaling	(114, 122)
		Enhancement of brain reparative microglial reactions by secreting SPP1 and through the ITGB1 signaling in the stroke mouse model	(106)
		Suppression of cytotoxic microglial factors NOX2 and iNOS through IL-4 mediated mechanism in ALS mouse model	(126)
		Improvement of cognitive function and disease pathology in APP/PS1 AD mouse model and MPTP-induced PD mouse model by inducing Tregs through suppression of microglial responses	(127, 129)
	Oligodendrocyte	Promoting oligodendrocyte differentiation through CCN3 signaling	(21)
	Neuron	Reduction of inflammatory cytokine expression and 5-TH dopaminergic neuron loss in MPTP induced-PD mouse model by adoptive transfer of Tregs	(136)
		Suppression of EAE disease progression by transferring of Tregs, co-cultured with neurons	(138)
	Stem cell	Induction of NSC proliferation in adult mouse SVZ through transplantation of active Tregs *via* IL-10 signaling	(143)
Intestine	External neuron (Vagus nerve)	Increased Tregs in mesenteric lymph node by vagus nerve stimulation but reduced Tregs and RORγt^+^ pTregs by vagotomy	(11, 158)
	Enteric neuron	Enhanced differentiation of Treg into RORγt^+^ Tregs by IL-6 secreted from ENS	(170)

As discussed in this review, the communication between the CNS, the GI tract, and the microbiota is essential for the exquisite control linking emotional and cognitive centers of the brain with peripheral intestinal/immunological functions, known as ‘gut-brain axis’. Although the mechanisms of this inter-organ interaction are now being elucidated, many aspects remain unknown. Neuronal innervations into the GI tract can regulate local immune responses through neuro- (neurotransmitters and neuropeptides) as well as immunological- (cytokines and chemokines) messengers. However, it is still primitive to elucidate the importance of neuroimmunological functions with detailed mechanisms in health and disease. Considering the differences in Tregs populations and proportion along the intestinal tract, one interesting question is whether neuronal innervation is directly or indirectly involved in this divergence and the neuro-/immunological meaning of this distinction (171). In the other way around, the immune cells in the GI tract, including Tregs, may also regulate the neurons innervating along the GI tract, which could modulate neuronal as well as intestinal functions (179, 180). Further understanding the regulation of gut-brain axis by Tregs may give a better comprehension of inter-organ communication between CNS and other organs. In a manner similar to the gut-brain axis, recent research reveals that microbial products in the lungs can likewise alter brain function and disease pathology, known as the lung-brain axis (181). This suggests that not just gut but multiple organs might be potentially communicated with neurological compartment for tissue homeostasis. Because Tregs are present in nearly all organs, the role of long-range communications for inter-tissue homeostasis and the manner in which Tregs engage in this process are important questions that must be addressed in the future.

## Author Contributions

LC, JC, B-RK, and BA wrote the manuscript and generated figures and tables. J-YL and H-KK edited the manuscript. All authors contributed to the article and approved the submitted version.

## Funding

This work was supported by a faculty research grant of Yonsei University College of Medicine (6-2021-0155), a grant of the Korea Health Technology R&D Project through the Korea Health Industry Development Institute (KHIDI), funded by the Ministry of Health & Welfare, Republic of Korea (HV21C0050, HV20C0172), and the Ministry of Education of the Republic of Korea and the National Research Foundation of Korea (2021R1C1C1006912, 2021R1A2C2004501, 2020R1I1A1A01069041, 2019R1A6A1A03032869).

## Conflict of Interest

H-KK is a scientific advisor member in Enhanced Neo Cell.

The remaining authors declare that the research was conducted in the absence of any commercial or financial relationships that could be construed as a potential conflict of interest.

## Publisher’s Note

All claims expressed in this article are solely those of the authors and do not necessarily represent those of their affiliated organizations, or those of the publisher, the editors and the reviewers. Any product that may be evaluated in this article, or claim that may be made by its manufacturer, is not guaranteed or endorsed by the publisher.
